# High-Density Real-Time PCR-Based *in Vivo* Toxicogenomic Screen to Predict Organ-Specific Toxicity

**DOI:** 10.3390/ijms12096116

**Published:** 2011-09-19

**Authors:** Gabriella Fabian, Nora Farago, Liliana Z. Feher, Lajos I. Nagy, Sandor Kulin, Klara Kitajka, Tamas Bito, Vilmos Tubak, Robert L. Katona, Laszlo Tiszlavicz, Laszlo G. Puskas

**Affiliations:** 1 Avicor Ltd., Közép fasor 52, Szeged H-6726, Hungary; E-Mail: gabriella@avicorbiotech.com; 2 Laboratory of Functional Genomics, Institute of Genetics, Biological Research Center, Hungarian Academy of Sciences, Temesvári krt. 62, Szeged H-6726, Hungary; E-Mails: farago.nora@gmail.com (N.F.); klarakitajka@gmail.com (K.K.); 3 Avidin Ltd., Közép fasor 52, Szeged H-6726, Hungary; E-Mails: l.feher@avidinbiotech.com (L.Z.F.); lajos@avidinbiotech.com (L.I.N.); kulinsandor@gmail.com (S.K.); 4 Obstetrics and Gynecology Department, Faculty of Medicine, University of Szeged, Semmelweis u. 1., Szeged H-6725, Hungary; E-Mail: bito@obgyn.szote.u-szeged.hu; 5 Curamach Ltd., Temesvári krt. 62, Szeged H-6726, Hungary; E-Mails: vili@brc.hu (V.T.); katona@brc.hu (R.L.K.); 6 Laboratory of Chromosome Structure and Function, Institute of Genetics, Biological Research Center, Hungarian Academy of Sciences, Temesvári krt. 62, Szeged H-6726, Hungary; 7 Department of Pathology, University of Szeged, Szeged H-6725, Hungary; E-Mail: tiszlats@patho.szote.u-szeged.hu

**Keywords:** toxicogenomics, organ toxicity, real-time PCR, gene expression

## Abstract

Toxicogenomics, based on the temporal effects of drugs on gene expression, is able to predict toxic effects earlier than traditional technologies by analyzing changes in genomic biomarkers that could precede subsequent protein translation and initiation of histological organ damage. In the present study our objective was to extend *in vivo* toxicogenomic screening from analyzing one or a few tissues to multiple organs, including heart, kidney, brain, liver and spleen. Nanocapillary quantitative real-time PCR (QRT-PCR) was used in the study, due to its higher throughput, sensitivity and reproducibility, and larger dynamic range compared to DNA microarray technologies. Based on previous data, 56 gene markers were selected coding for proteins with different functions, such as proteins for acute phase response, inflammation, oxidative stress, metabolic processes, heat-shock response, cell cycle/apoptosis regulation and enzymes which are involved in detoxification. Some of the marker genes are specific to certain organs, and some of them are general indicators of toxicity in multiple organs. Utility of the nanocapillary QRT-PCR platform was demonstrated by screening different references, as well as discovery of drug-like compounds for their gene expression profiles in different organs of treated mice in an acute experiment. For each compound, 896 QRT-PCR were done: four organs were used from each of the treated four animals to monitor the relative expression of 56 genes. Based on expression data of the discovery gene set of toxicology biomarkers the cardio- and nephrotoxicity of doxorubicin and sulfasalazin, the hepato- and nephrotoxicity of rotenone, dihydrocoumarin and aniline, and the liver toxicity of 2,4-diaminotoluene could be confirmed. The acute heart and kidney toxicity of the active metabolite SN-38 from its less toxic prodrug, irinotecan could be differentiated, and two novel gene markers for hormone replacement therapy were identified, namely *fabp4* and *pparg*, which were down-regulated by estradiol treatment.

## 1. Introduction

Gene expression profiling of drug or xenobiotic exposed cells or animals is rapidly becoming a standard analysis in toxicology, and has the potential to play a pivotal role in all stages of drug safety evaluation including preclinical and clinical studies. Toxicogenomics is an emerging technology that uses novel genomic technologies to investigate the adverse effects of small molecules at the transcriptome level [1–3]. Among the applied technologies, DNA microarrays and new generation sequencing methods have the capability to screen drug-induced gene expression changes at a global scale [4,5].

These up-to-date technologies are allowing researchers to gain an increased understanding of the function and regulation of genes and to identify pathways that are affected.

Toxicogenomics is based on the fact that most relevant toxicological effects of a compound affect directly or indirectly the gene expression. In order to demonstrate that different mechanisms of toxicity can be determined from gene expression data, Dai *et al*. have analyzed expression profiles of samples from rodents treated with 49 known hepatotoxins and 10 compounds without known liver damage. By using their bioinformatic tools compound-induced liver toxicity could be predicted with 90.9% sensitivity and 88.4% specificity [6]. In another study Hamadeh *et al*. tested the hypothesis that cDNA microarrays are an applicable platform for chemical-specific gene-expression profiling [7]. Relative expression changes were clustered and correlated to histopathology and chemical data, which corresponded well. Thus, it may be possible to determine if the compound has potential toxicity by comparing the gene expression profiles of an unknown compound against a reference database.

The most important advantage of toxicogenomics is the early predictive capability based on the temporal effects of drugs on gene expression: changes in genomic biomarkers may occur before subsequent protein translation and initiation of histological organ damage. Most of the previous studies focused on only one tissue or organ, such as liver [8–11], kidney [12], spleen [13], lung [14], brain [15], or one type of toxic insult, such as genotoxicity and carcinogenecity [16] or phospholipidosis [17].

In the present study our objective was to extend *in vivo* toxicogenomic screening from analyzing one or a few tissues to multiple organs. Because of the biological variation of the tested animals in our standard protocol eight animals were used (4 treated and 4 buffer-treated controls). From each animal, four different organs (liver, kidney, heart and brain) were collected to study their gene expression profiles.

Although DNA microarray technology became a powerful screening tool for gene expression profiling in toxicogenomics, not only sensitivity and dynamic range are small, but in our set-up screening of each drug candidate would need 32 microarrays. Therefore, when multiple samples are intended to be analyzed for organ-specific toxicity, application of DNA microarrays are technically challenging and expensive.

Moreover, standardization of data analysis and comparison can be difficult because of different platforms available. Quantitative real-time PCR (QRT-PCR) remains one of the gold standards in accurate determination of gene expression changes and has been already applied to validate microarray data in toxicogenomic studies and for molecular phenotyping [18–21].

The high sensitivity, reproducibility, and large dynamic range of traditional QRT-PCR provides high-throughput and accurate differential expression profiling of usually 10–20 selected genes. However, one of the drawbacks of application of traditional QRT-PCR in toxicogenomics is the relatively low throughput and the small number of genes that can be analyzed on multiple samples.

Recently, a novel, nanocapillary-based QRT-PCR has been established with a capacity of running approximately 18,000 reactions per day in one OpenArray^™^ Cycler (Biotrove, Applied Biosystems). The system runs with high accuracy, precision and provides dynamic range characteristic of QRT-PCR with the relatively higher throughput of microarrays: 3072 individual solution-phase reactions are run parallel in 33 nL through-holes on the size of a microscope slide in a thermal cycler. This platform is optimal for analyzing numerous samples over 56–112 gene markers. Previously the analytical performance of this technology and its general applicability for the field of toxicogenomics was confirmed by screening 668 compounds for their gene expression profiles *in vitro* in HepG2 cells [2]. In this study we have also demonstrated that a focused set of marker genes can be used for finding the correlation between a library of molecular scaffolds and their general biological fingerprint.

In the present study, reference as well as discovery drug-like compounds were screened for their gene expression profiles in different organs of treated mice over a discovery gene set of 56 toxicology biomarkers. Marker genes were selected from DNA-microarray and literature data that cover different pathways altered during toxic insults in the brain, lungs, spleen, heart, liver and kidney. We demonstrated that using high-throughput QRT-PCR technology for *in vivo* toxicogenomic study of different organs from treated animals can be used for preclinical studies and could accurately predict organ-specific toxic side effects.

## 2. Results and Discussion

### 2.1. Results

#### 2.1.1. Development of a Toxicogenomic Nanocapillary QRT-PCR Platform

Although DNA-microarray technology is able to determine the expression of virtually all genes in the genome, application of this approach in a medium-throughput screening project is labor and material intensive and it generates overwhelming data with no predictive value on toxic side effects. Therefore it is feasible to identify a smaller number of genes that may serve as selective markers for early toxicogenomic screening. Previously we demonstrated the utility of high-throughput, nanocapillary QRT-PCR system, which uses the OpenArray^™^ Cycler from Applied Biosystems (previously BioTrove) [2]. It joins high accuracy, precision and dynamic range characteristic of QRT-PCR with the higher throughput of microarrays: 3072 individual solution-phase reactions are running in parallel in a matrix of 48 submatrices having 64 through-holes in each (out of which 56 can be used for gene expression profiling). Low sample-volumes (33 nL in each hole), high number of reaction chambers; the 48 individually addressable submatrices and the software-controlled data processing and analysis make the system ideal for toxicogenomic screening. Because of the characteristics of the nanocapillary system definition of 56 genes (or 2 × 56 genes) are optimal for large scale toxicogenomic analysis. By selecting 56 genes one can analyze up-to 144 samples per run. Because of the high sample number we designed our toxicogenomic platform to be able to determine organ-specific toxicity. Accordingly, we selected 56 genes from DNA-microarray and literature data that cover different pathways altered during toxic insults in different organs: the brain, lungs, spleen, heart, liver and kidney. The list of the selected genes, the organs that have been correlated with their toxic effects and the references are shown in Table 1. The selected genes can be classified by their functions: they code for proteins for acute phase response (*saa3*, *anxa2*, *fga*, *ftl1*), inflammation (*tubb5*, *reg3a*, *serpine1*, *fabp4*, *serpinci*, *fas*), oxidative stress (*gadd153*, *nox3*, *ldh3b*, *prdx3*, *alox12b*, *akr1b8*, *prdx1*, *sod1*, *nqo1*, *cfos*), metabolic processes (*oazi*, *timp3*, *pepck*, *hsd3b4*, *odc1*, *kap*, *rbp4*, *aadat*, *pgam2*, *ndufa5*, *ptpmt1*, *timp2*, *klk1b3*), heat-shock response (*dnaja2*, *hspcb*, *hspa1a*), cell cycle/apoptosis regulation (*clu*, *spp1*, *vim*, *ccng1*, *egf*, *psmb8*, *ubc*, *pcna*) and enzymes which are involved in detoxification (*gstp2*, *oat*, *hsd17b4*, *cyp1a1*, *cyp7a1*, *ephx1*, *slc25a6*). Three housekeeping genes were selected (*ppia*, *pgk1* and *rplp0*) and their average expression was used for normalization.

Because of the relatively high number of animals needed for our toxicogenomic platform we designed our gene sets for mouse; however the same set of genes could be designed for other organisms as well, such as for rat or rabbit. The discovery set of gene markers presented here can be also further optimized and could be revised in each order of the OpenArray^™^ plates.

#### 2.1.2. *In Vivo* Protocol for Toxicogenomic Profiling of Multiple Organs

In the present study our objective was to extend *in vivo* toxicogenomic screening from analyzing one or a few tissues to multiple organs. Because of individual differences of the tested animals, four animals are used in each group treated either with control solubilization buffer or with a toxic reference or a drug-like compound. In the present study four different organs (liver, kidney, heart and brain) were isolated from each animal to study their gene expression profiles. However, other organs, such as lungs, spleen, testis, ovary can be also analyzed with our technology, as several gene markers overlap between different organs. Marker gene design was also based on previous studies on different cells and tissues (Table 1), moreover other new markers could also be inserted into our list, therefore adaptation of the high-throughput QRT-PCR for analyzing virtually any tissues can easily be done.

Based on the expression data from six hepatotoxins in rat livers obtained on DNA microarray at multiple time-points, Bulera *et al*. found that the expression profiles from the same compounds clustered together regardless of treatment duration [33]. This indicates that individual compounds give unique expression signatures and the treatment duration can be standardized in a toxicogenomic study. Different tested compounds were applied for 16 h and brains, hearts, kidneys and livers were isolated afterwards. Because of the different solubility of the tested compounds a solubilization procedure was used, which successfully increased the solubility of different compounds. The compounds were dissolved in DMSO, then a non-ionic detergent, Solutol (BASF, Germany) was added and finally saline was used to obtain a clear solution, which could be injected intraperitoneally. This administration route was used to determine systemic effects and to avoid differences in bioavailibility of the different drugs. After organ isolation, RNA was stabilized in RNA-Later (Ambion, Life Technologies, USA) at 4 °C for 16 h. After RNA purification and cDNA conversion the templates were applied to each sub-matrix of the OpenArray^™^ plate. In one run four different organs from animals of three different treatments and one control group could be analyzed in quadruplicates (biological replicates).

**Figure 1:**
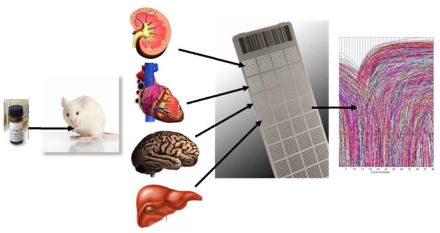
Schematic representation of our organ-specific toxicogenomic screening based on nanocapillary QRT-PCR technology.

The schematic representation of the present protocol can be seen in Figure 1. Although one concentration from each tested compound was used based on known LD_50_ data, one can analyze different concentrations of the same drug to determine safe dosing of the drug candidate by simply analyzing the gene expression profiles.

#### 2.1.3. Profiling of Known Toxic Reference Compounds

**Figure 2:**
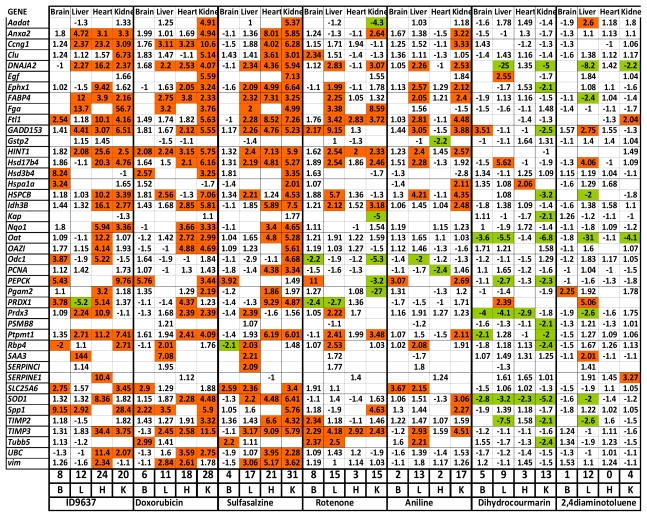
Marker gene expression profiles of different reference toxic compounds. Differences are given in average ΔCt values after normalization to housekeeping genes. Green labels indicate repression, while red labels indicate induction of gene expression activity, compared to vehicle treated animals. For each treatment and for each organ we calculated the number of genes affected (induced or repressed) (see below in each column).

To test our toxicogenomic profiling approach, we determined how known toxic reference compounds affect the expression of the selected marker genes and whether we can record organ-specific alterations based on the expression profiles. At first in a verification study doxorubicin, sulfasalazin, rotenone, aniline, dihydrocoumarin and 2,4-diaminotoluene was injected into mice in the same carrier solution (20% DMSO, 25% Solutol, 55% saline) and compared the expression of genes to those obtained from animals having the same carrier solution with no compounds. After injecting four animals with each compound intraperitoneally, four organs were collected: heart, brain, liver and kidney and they were subjected to mRNA purification, cDNA conversion and nanocapillary QRT-PCR. After data analysis different gene expression changes were found in response to different chemicals, moreover organ-specific changes for each toxic compound could be recorded (Figure 2).

In case of doxorubicin treatment at 20 mg/kg dose, most of the induced genes could occur in the heart and the kidney which is in good concordance with the known toxicity of this chemotherapeutic agent [34]. To test concentration dependent changes, doxorubicin was applied at lower concentration as well (5 mg/kg). As expected at lower doses smaller number of genes were affected (3 *vs.* 6 in the brain, 11 *vs.* 28 in the kidney, 2 *vs.* 18 in the heart, and 8 out of 11 in the liver). Moreover, at lower doses almost all genes that showed more than 2-fold gene expression alteration were similar to those that exhibited significant changes at higher doses (data not shown).

High toxicity of i.p. administered sulfasalzine, a widely used anti-inflammatory agent, could be registered based on its dramatic effects on gene expression in all organs, except in the brain, which might be due to its lower toxicity at this concentration or lower penetration ability through the blood brain barrier. Rotenone, a pesticide and mitochondrial complex I inhibitor, caused general toxicity when administered into animals [35]. Interestingly, although rotenone induces oxidative stress, we could register brain, liver and kidney toxicity and no, or very slight cardiotoxicity based on the number of genes altered in our study (Figure 2). Similarly, aniline and dihydrocoumarin induced marker gene expression changes in the liver and kidney, however they resulted in completely different gene expression profiles: aniline induced 16 gene markers, while dihydrocoumarin down-regulated 10 genes, out of which 6 were in common with those affected by aniline. 2,4-diaminotoluene, a known hepatotoxic agent [36], resulted in a very specific hepatotoxic gene expression signature and induced hardly any changes in gene expression in the other three organs.

#### 2.1.4. Profiling of Drugs and Prodrugs

For our in-house drug discovery program, we used our toxicogenomic screen for early prediction of side effects. Mice were treated with 30 mg/kg Ac-915, a novel lipid-droplet binding thalidomide analog [37] and with 20 mg/kg ID9637, a fatty acid derivative, as a novel anticancer drug candidate [38]. By using the QRT-PCR profiling of different organs of the treated animals, Ac-915 resulted in 3 induced genes in brain samples, 1 repressed gene in the heart, 9 induced genes in the liver and 10 induced genes in the heart (data not shown). ID9637 caused massive gene expression alterations in all of the tested organs (brain: 8 genes, heart: 24 genes, liver: 12 genes and kidney: 20 genes) (Figure 2). These results suggested that at these concentrations these compounds are highly toxic, which was well correlated by classical toxicology end-point results.

In our toxicogenomic test we also studied Trisequens N, which is a hormone replacement therapy preparation. It consists of estradiol hemihydrate alone and in combination with norethisterone acetate. Estradiol hemihydrate is a naturally occurring form of estrogen and norethisterone acetate is a synthetic form of progesterone [39]. By applying these drugs to mice we detected minor changes in gene expression. In the brain only one gene was repressed (*spp1*) by the estradiol treatment and one was induced (*timp2*) by oestrogen and norethisterone treatment. Similarly in the heart the expression of only one gene was elevated in each case (*skp2* for estradiol alone and *tubb5* for the combination treatment), in the liver one gene was repressed by both treatments (*ldh3b*) and one additional induction occurred in response to estradiol (*Slc25a17*). In the kidney the expression of *gclc* was up-regulated in animals receiving the drug combination, while in both treatment groups two additional genes were repressed (*fabp4* and *pparg*). These changes are minor and could represent individual deviations and most probably they do not account for toxic side effects.

We intended to apply our toxicogenomic platform to see whether the toxic activities of a prodrug (irinotecan) and its active metabolite (7-ethyl-10-hydroxycamptothecin, SN-38) could be differentiated. Heart, brain, kidney and liver were dissected from 4-4 treated animals and gene expression profiling was done with nanocapillary QRT-PCR over a discovery gene set of 56 toxicology biomarkers. A single gene was induced by irinotecan (*hspcb* by 4.72-fold) out of 56 genes examined in the brain samples, while SN-38 treatment resulted in one repressed (*c-fos* by 2.39-fold) and four induced genes (*skp2*: 2.5-fold; *vim*: 2.28-fold; *nqo1*:3.14-fold; *sod1*: 4.76-fold).

In the kidney 5 and 7 genes were affected by irinotecan and SN-38, respectively. All of the 5 genes which were up-regulated in response to irinotecan were found to be induced as well, in the SN-38 treated samples. These genes were the following: *gclc*: 3.39-fold and 5.70-fold; *pepck*: 2.91-fold and 3.43-fold; *odc1*: 2.77-fold and 4.38-fold; *fabp4*: 2.75-fold and 5.28-fold; *pparg*: 3.07-fold and 3.03-fold, in the irinotecan and SN-38 treated kidney tissues, respectively. Two genes were induced only in the SN-38 treated group: *hspa1a* by 3.14-fold and *fga* by 3.01-fold.

In the liver more striking difference could be observed between the two groups: irinotecan induced one gene (*kap* by 3.6-fold) and down-regulated one gene (*clu* by 2.2-fold), while SN-38 treatment elevated the mRNA level of 5 genes (*serpine1* by 5.58-fold, *skp2* by 2.69-fold, *pgam2* by 2.51-fold, *gadd153* by 3.78-fold and *trp2* by 16-fold).

In the heart 2 genes were repressed (*odc1* by 2.04-fold and *ccng1* by 2.91) and one was induced (*prdx1* by 2.33-fold) in response to irinotecan, while altogether the expression of 8 marker genes were affected by SN-38 (induced: *odc1* by 2.56-fold, *prdx1* by 2.58-fold, *ftl1* by 8.28-fold, *tubb5* by 2.56-fold, *gpx4* by 2.97-fold, *ccng1* by 2.69; repressed: *kap* by 2.89-fold and *hint1* by 5.39-fold).

#### 2.1.5. Comparison of Data from Toxicogenomic and Histological Analysis

To test whether toxicology-related gene expression changes can be correlated with pathological observations on histological samples, we selected two treated groups from those samples where significant number of altered genes could be registered.

Histological sectioning and analysis from groups treated with ID9637 and sulfasalazin were performed. In the brain, heart and kidney we could not observe any histological changes in the treated groups. Any signs of toxic side effects could be observed in other organs (spleen, lungs and liver) from the sulfasalazin group (see pictures in Supplementary Figure 1). In the liver the ID9637 treated animals’ medium portal-periportal inflammation and gathered fibrin-pus in the fibrotic liver capsule could be observed (see pictures in Supplementary Figure 2).

### 2.2. Discussion

During the drug developmental process, undesired toxicity accounts for about one third of compound failures [40]. However, hepatotoxicity is a common reason for withdrawal of compounds from the market [41], drug-induced toxicity affecting other organs, including kidney, heart and the central nervous system, is a common finding in the preclinical phase of drug development [39,42]. Therefore, it is evident that new technologies are needed as an alternative to classical toxicological tests for prediction of side effects specific to different organs. Toxicogenomics is an emerging technology that uses novel genomic methods to investigate the adverse effects of small molecules at the transcriptome level including DNA microarrays, new generation sequencing and QRT-PCR methods [1–3,5,20]. Among the applied technologies traditional QRT-PCR provides high-throughput and accurate differential expression profiling of usually 10–20 selected genes with high sensitivity, reproducibility, and large dynamic range. However, one of the drawbacks to apply traditional QRT-PCR in toxicogenomics is the relatively low throughput and the small number of genes that can be analyzed on multiple samples simultaneously. Because of the relatively high number of samples that are required to be analyzed and because of the better predictive value of larger gene sets (50–100 genes) for organ-specific toxicity, a high-throughput QRT-PCR approach is needed. Previously we confirmed the analytical performance of a novel, nanocapillary-based QRT-PCR, the OpenArray^™^ system (Applied Biosystems, previously Biotrove Inc.) for toxicogenomic screening of 668 compounds for their gene expression profiles in HepG2 cells [2]. This high-throughput QRT-PCR has a capacity of running about 18,000 reactions per day and it is optimal for analyzing numerous samples over 56–112 gene markers.

It is clear that there are a number of limitations using *in vitro* approaches such as the functional differences observed in primary cells relative to the intact organs, the absence of interactions with biological borders and matrices (*i.e.*, for ADME effects) under *in vitro* conditions, which are representative of an *in vivo* situation. Therefore, our objective was to develop an *in vivo* toxicogenomic screening to analyze multiple organs after systemic administration of the tested compound. Because of the relatively high number of samples needed for our test (multiple organs from numerous biological replicates) the OpenArray^™^ platform was adopted to determine relative changes in expression of 56 toxicology-related genes.

Based on our previous, and on literature, data from DNA-microarray experiments, 56 gene markers were selected coding for proteins having roles in acute phase response, inflammation, oxidative stress, metabolic processes, heat-shock response, cell cycle/apoptosis regulation and detoxification. The ideology of gene selection was that transcriptional regulation of the genes should be observed in response to drug treatment that had been suggested as markers for early stages of toxic effects, therefore they could be used as predictive markers. Some of the genes are induced upon xenobiotic or toxic compounds in a specific organ, while others are general indicators of toxicity of multiple organs. Previous studies showed that gene expression profiling of samples having isolated at multiple time-points resulted in very similar alterations to control samples regardless of treatment duration [33]. Based on this observation we applied different tested compounds for 16 h and brains, hearts, kidneys and livers were isolated afterwards. Mouse was used as model organism for our toxicogenomic study because relatively high number of animals is needed. For QRT-PCR we used Taqman chemistry, instead of SybrGreen protocol. Taqman probes were designed for mouse genes; however the same set of genes could be designed for other organisms as well. Because of the open design of the OpenArray^™^ plates our discovery gene set can be further optimized having novel genes inserted or replacing genes responding only in very specific cases. The number of selected marker genes can be increased and specific genes can be inserted, especially if the mechanism of action is known for a compound and other pathways are affected.

To test our toxicogenomic screening platform relative gene expression changes after systemic administration of known toxic reference compounds (doxorubicin, sulfasalazin, rotenone, aniline, dihydrocoumarin and 2,4-diaminotoluene) were determined. The number of modulated genes differed between the various treatments. The more genes there were affected by a compound in a certain organ, the more toxic effects could be verified.

In case of doxorubicin treatment most of the induced genes could occur in the heart and the kidney which is in good concordance with the known toxicity of this chemotherapeutic agent. The most important cardiotoxic mechanisms proposed for doxorubicin include oxidative stress with its resultant damage to myocardial elements, changes in calcium homeostasis and decreased ability to produce ATP [34]. In our study out of 10 genes that are involved in the oxidative stress response six were induced in the heart and five in the kidney (Figure 2). When doxorubicin was applied at two different concentrations dose-depentend gene expression alteration could be detected.

Sulfasalazine is a drug commonly used in the treatment of inflammatory bowel diseases such as ulcerative colitis and Crohn’s disease and rheumatoid arthritis. Frequent incidence of side effects limits therapy with sulfasalazine, which is due to its effects on oxidative stress [43]. Sulfasalazine induced dramatic changes in the expression of marker genes in the liver, in the kidney and in the heart suggesting severe toxicity when systemically applied at high doses.

Rotenone, a pesticide and mitochondrial complex I inhibitor, triggers general toxicity when administered to animals [35]. Interestingly, brain, liver and kidney toxicity could be registered and no or very slight cardiotoxicity based on the number of genes altered in the study (Figure 2). In case of aniline and dihydrocoumarin we found substantial gene expression modification in the liver and kidney, however they generated completely different gene expression profiles: aniline induced 16 gene markers, while dihydrocoumarin down-regulated 10 genes, out of which 6 were in common with those affected by aniline. Genes specifically altered by treatment of 2,4-diaminotoluene, which induce DNA damage, DNA repair and micronucleus formation in hepatoma cells [36], could be detected in the liver and not in any other organs.

From these results we could conclude that based on early gene expression changes the present genomic approach is able to predict organ-specific transcriptional response.

To demonstrate the utility of the strategy different drugs and drug candidates were profiled. In in-house anticancer drug discovery programs at Avidin we were able to demonstrate the high toxicity of a fatty acid derivative cytotoxic agent, ID9637 and cardio- and hepatotoxicity of a novel lipid-droplet binding thalidomide analog [37].

However, gene expression changes may represent organ adaptation to chemical exposure without acute toxicity. The advantage of toxicogenomic screen over classical methods is to identify genetic elements that could be correlated and even to predict toxic insult when there is still no pathological readout. If a compound induces several genes that are part of the organ adaptation, one could expect organ toxicity or induced activity, which could end in organ failure and organ toxicity upon chronic administration of the drug. Histological analysis was performed from those samples where significant number of altered genes could be registered (in case of ID9637 and sulfasalazin). Although in the sulfasalazin treated organs, a high number of toxicology-related genes were induced, no pathological alteration could be observed in the brain, heart, kidney, spleen, lungs and liver. In the ID9637 treated animals, no signs of toxic effects could be observed in the histological sections of the organs, except the liver, where acute toxic side effects could be registered (medium portal-periportal inflammation and gathered fibrin-pus in the fibrotic liver capsule). From these results we could conclude that early gene expression changes cannot be accurately compared with pathological alterations, mainly because of the different time scale of the methods.

In the present toxicogenomic test Trisequens N was also studied, which is a hormone replacement therapy preparation. It consists of estradiol hemihydrate alone and in combination of norethisterone acetate. These ingredients are forms of the main female sex hormones, estrogen and progesterone [39]. Estradiol hemihydrate is a naturally occurring form of estrogen and norethisterone acetate is a synthetic form of progesterone. By applying these drugs to mice we were interested in whether a single injection results in any changes in the expression of our marker genes. No significant changes between the treated and the control groups were found. Although very small changes in the model of hormone replacement therapy were registered, interestingly, in the kidney down-regulation of both *fabp4* and its transcription regulator, *pparg* were detected. FABP4 is a lipid binding protein playing a role in intracellular lipid transport and metabolism, as well as in signal transduction and its expression is regulated by PPAR-dependent transcriptional mechanism [44]. Both gene products are associated with metabolic syndrome, type 2 diabetes mellitus, cancer and atherosclerosis [44–46]. Although in the acute experiment any significant changes could be found in the expression profiles of the treated animals, further studies on the expression alteration of *fabp4*, *pparg* or other gene markers could possibly highlight the effects of chronic hormone replacement therapy applied at different doses and could define the risk population.

Irinotecan, a widely used chemotherapeutic agent is activated by hydrolysis to SN-38, an inhibitor of topoisomerase I. The inhibition of this enzyme by the active metabolite SN-38 leads to inhibition of both DNA replication and transcription and finally apoptosis of cancer cells. Previously Blandizzi and his co-workers described that the antitumor drug irinotecan possesses adverse cardiovascular effects [47], while the same drug was demonstrated to have negative effects on renal functions [48]. The present toxicogenomic platform was applied in order to demonstrate whether the toxic activities of irinotecan and its active metabolite 7-ethyl-10-hydroxycamptothecin (SN-38) could be differentiated. As irinotecan is a prodrug, it was hypothesized that it has less influence on gene expression of toxic markers than SN-38, when applied at the same concentrations.

Gene expression screening results indicate that SN-38 exerts negative effects on both heart and kidney, as determined by altered toxic gene marker expression. Similar effects could be seen in case of irinotecan; however a smaller number of genes were affected. This observation is in good concordance with the different tolerability of the prodrug and the drug. Our results demonstrate that the presented toxicogenomic platform is not only able to detect organ-specific transcriptional response of different harmful chemicals, but also able to distinguish the toxic effects of a prodrug and its active metabolite.

One of the limitations of our gene selection procedure is that although some of the genes might be useful indicators of toxicity in a specific organ, in some cases they cannot be used in other organs. Some genes showed lower, or even undetectable expression by nanocapillary QRT-PCR (e.g., in case of *egf*, *serpinCI*, *saa3*, *kap* or *serpinEI*) in some tissues. This organ-specific difference was more pronounced in the brain, where the lesser number of genes showed altered expression, even in those cases where toxicity of the brain could be predicted. This problem could be overcome with precise selection of marker genes in the future and with developing an improved version of nanocapillary QRT-PCR toxicogenomic platform.

## 3. Experimental Section

### 3.1. Animals, Treatment, and Sample Collection

Groups of 4 Balb/C female mice, that were kept in a conventional animal house and received conventional food pellets and tap water *ad libitum* throughout the experiments, were injected intraperitoneally with 400 μL carrier solution (20% DMSO, 25% Solutol (BASF, Germany), 55% saline) for control, or compound dissolved in 400 μL carrier solution in the following doses: doxorubicin (Sigma-Aldrich, Budapest, Hungary): 5 mg/kg and 20 mg/kg; rotenone (Sigma-Aldrich): 30 mg/kg; aniline (Sigma-Aldrich): 150 mg/kg; sulfasalazin: 30 mg/kg; dihydrocoumarin (Sigma-Aldrich): 80 mg/kg; 2,4-diaminotoluene (Sigma-Aldrich): 80 mg/kg; irinotecan (Sigma-Aldrich): 10 mg/kg; SN-38 (Sigma-Aldrich): 10 mg/kg; Ac-915 (Avidin, Szeged, Hungary): 30 mg/kg; ID9637 (Avidin): 20 mg/kg; estradiol: 200 μg/kg and combination of 200 μg/kg estradiol and 100 μg/kg norethisterone (Novo Nordisk, Bagsværd, Denmark). After 16 h brains, hearts, kidneys and livers were isolated and stored in RNA-Later (Ambion, USA) at 4 °C overnight. All animal experiments were performed respecting institutional animal welfare guidelines.

### 3.2. RNA Isolation

RNA isolation from heart tissue was performed as published [49]. Briefly, our protocol is an improved version of the High Pure miRNA Isolation Kit (Roche, Cat. No. 05080576001) with inserting several additional steps into the standard protocol. Mouse hearts were frozen and homogenized at the temperature of liquid nitrogen. To 50 mg tissue powder 190 μL proteinase K solution was added (prepared as follows: 120 μL Paraffin Tissue Lysis Buffer (Roche, Germany), 20 μL 10% SDS and 50 μL Proteinase K (Roche). Samples were incubated at 55 °C for 30 min. After incubation 325 μL Binding Buffer (Roche) and 320 μL Binding Enhancer (Roche) was added and loaded onto the filter columns (Roche). Next the filters were washed in two steps with 500 and 300 μL of Washing Buffer (Roche) then the RNA was eluted by adding 40 μL Elution Buffer (Roche). The quality and quantity was assessed spectrophotometrically (Nanodrop, USA) and considered acceptable if the absorption ratio of 260/280 was >1.8.

Brain tissue was homogenized in Trizol reagent (Sigma), liver and kidney was homogenized in RA1 buffer (Machery-Nagel, USA). Total RNA was purified from drug treated and control organs with AccuPrep^™^ RNA purification kit (Bioneer, Daeleon, Korea) according to the manufacturers’ protocol, except that DNase I treatment was incorporated. Homogenized tissues were centrifuged through NucleoSpin Filters (Machery-Nagel 740606, 13,000 rpm, 3 min in Eppendorf centrifuge). Pellet was suspended in RA-1 lysis buffer (Machery-Nagel, 740961.500) supplemented with β-mercaptoethanol. Equal volume of 70% ethanol was also added, samples were vortexed, and were loaded onto extraction columns (Bioneer Viral RNA Extraction Kit, KA-1111). Columns were centrifuged with 13,000 rpm, 1 min in Eppendorf centrifuge, washed with 80% ethanol, than treated with DNase for 15 min at RT. Reaction was stopped with RA1:EtOH (1:1), centrifuged, then washed twice with Wash Buffer 2 (Bioneer, KB1052). RNA was eluted with 50 μL RNase free water at 55 °C, and the concentration was determined by Nanodrop. After addition of RNase inhibitor, samples were stored at −80 °C.

For QRT-PCR total RNA (750 ng) was converted into cDNA with the High-Capacity cDNA RT Kit (Applied Biosystems, Foster City, CA, USA) and without purification the mixture was diluted with RNase-free water and applied to QRT-PCR analysis.

### 3.3. Profiling of RNAs with High-Throughput, Nanocapillary QRT-PCR

Amplification of the samples was followed in real time with an OpenArray NT Cycler (BioTrove Inc., Woburn, MA; Applied Biosystems, Foster City, CA, USA). For our discovery gene set individual Taqman assays were specified (Table 1). An aliquot of each Taqman assay was sent to BioTrove (Woburn, MA, USA) for loading in their OpenArray plates. Taqman assays are purchased individually and loaded by BioTrove (now at Applied Biosystems, Life Technologies) in a customer-specified layout. Recently, the list of the genes is available to prepare the custom-designed plates by the company. A third fluorescent dye (ROX), present in the Taqman assay mixture, was imaged to provide quality assessment of manufacturing and loading of the arrays.

The reverse transcribed samples (or water for no template controls) were added to a 384-well plate containing GenAmp Fast PCR Master Mix (Applied BioSystems, Foster City, CA, USA) and OpenArray DLP 5× Remix Solution (BioTrove Inc., Woburn, MA, USA) for OpenArray amplification. The OpenArray autoloader transfers the cDNA/master mix from the plate to the array through-holes by capillary action. Each subarray was loaded with 5.0 μL of master mix containing 1.2 μL of reverse transcribed cDNA. The array is manually transferred to the OpenArray slide case and sealed. The plates were cycled in the OpenArray NT cycler (up to three arrays simultaneously) under the following conditions: 50 °C for 15 s, 91 °C for 10 min, followed by 50 cycles of 54 °C for 170 s and 92 °C for 45 s.

The Biotrove OpenArray NT Cycler System software (version 1.0.2) uses a proprietary calling algorithm that estimates the quality of each individual threshold cycle (*C_T_*) value by calculating a *C_T_* confidence value for the amplification reaction. In our assay, *C_T_* values with *C_T_* confidence values below 300 (average *C_T_* confidence of the non-target amplification reactions plus 3 standard deviations) were considered background signals. Higher *C_T_* confidence levels were considered positive and were analyzed further. Normalization was done by using the average *C_T_* values of three house-keeping genes (*ppia*, *pgk1* and *rplp0*) and gene expression changes were calculated from the average of four replica experiments. Average values were accepted when the STD was below 0.5-fold of the average.

## 4. Conclusions

Here we report the application of a high-throughput, nanocapillary QRT-PCR-based toxicogenomic method to an *in vivo* organ-specific assay for cost-effective and robust testing of compounds. Although only a small set of known toxic chemicals was tested, our findings were in good correlation with previous toxicology studies. Besides verification data, we applied our strategy to drugs, drug candidates and prodrugs, which provided novel marker gene expression changes. These unique fingerprints underline the importance of expression profiling of a focused set of genes on different organs and warrant further development and full validation of such an alternative testing strategy for preclinical and environmental toxicology.

## Supplementary Information



## Figures and Tables

**Table 1:** List of marker genes, their references and Taqman probes used in this study.

#	Gene	Name	Accession No.	Probe Name	Organ	Ref.
**1**	GADD153	DNA-damage-inducible 3	NM_007837.3	Mm00492097_m1	liver	[7,21]
**2**	SAA3	serum amyloid A 3	NM_011315.3	Mm00441203_m1	liver, lung	[22]
**3**	TIMP3	metallopeptidase inhibitor 3	NM_011595.2	Mm00441827_m1	liver, lung	[22]
**4**	PEPCK	phosphoenolpyr. carboxykinase	NM_011044.2	Mm00440636_m1	liver	[5]
**5**	NOX3	NADPH oxidase 3	NM_198958.2	Mm01339132_m1	kidney	[6]
**6**	Hsd3b4	hydroxy-d-5-steroid dehyd.	NM_001111336	Mm00843753_s1	liver	[7]
**7**	Clu	clusterin	NM_013492.2	Mm00442773_m1	kidney, liver	[7,11]
**8**	Spp1	secreted phosphoprotein 1	NM_001204201	Mm00436767_m1	kidney	[11]
**9**	vim	vimentin	NM_011701	Mm01333430_m1	kidney	[11]
**10**	Anxa2	annexin A2	NM_007585.3	Mm00500307_m1	kidney	[23]
**11**	Tubb5	tubulin, beta 5	NM_011655.5	Mm00495804_m1	kidney	[11]
**12**	Gstp2	glutathione S-transferase, pi 2	NM_181796.2	Mm00839138_g1	kidney	[7]
**13**	Fga	fibrinogen alpha chain	NM_001111048	Mm00802584_m1	kidney	[24]
**14**	Ccng1	cyclin G1	NM_009831.2	Mm00438084_m1	kidney	[11]
**15**	Klk1b3	kallikrein 1-related peptidase b3	NM_008693.2	Mm01203825_gH	kidney	[11]
**16**	Odc1	ornithine decarboxylase 1	NM_013614.2	Mm01964631_g1	kidney	[11]
**17**	Kap	kidney androgen regulated prot.	NM_010594.2	Mm00495104_m1	kidney	[11]
**18**	Oat	ornithine aminotransferase	NM_016978.2	Mm00497544_m1	kidney	[11]
**19**	Rbp4	retinol binding protein 4	NM_001159487	Mm00803266_m1	kidney	[11]
**20**	Aadat	aminoadipate aminotransferase	NM_011834.2	Mm00496169_m1	kidney	[11]
**21**	Egf	epidermal growth factor	NM_010113.3	Mm01316968_m1	kidney	[11]
**22**	Pgam2	phosphoglycerate mutase 2	NM_018870.3	Mm00450782_g1	heart	[25]
**23**	Hsd17b4	hydroxysteroid dehydrogenase 4	NM_008292.4	Mm00500443_m1	heart	[25]
**24**	Idh3B	isocitrate dehydrogenase 3 beta	NM_130884.4	Mm00504589_m1	heart	[25]
**25**	Ndufa5	NADH dehydrogenase 1 alpha 5	NM_026614.2	Mm00471676_g1	heart	[25]
**26**	Prdx3	peroxiredoxin 3	NM_007452.2	Mm00545848_m1	heart	[25]
**27**	Alox12b	arachidonate 12-lipoxygenase	NM_009659.2	Mm00507782_m1	heart, brain	[26]
**28**	Reg3a	regenerating islet-derived 3a	NM_011259.1	Mm00441121_m1	heart, liver	[27]
**29**	Cyp1a1	cytochrome P450, family 1a1	NM_001136059	Mm00487218_m1	liver	[7]
**30**	SERPINE1	serine peptidase inhibitor E1	NM_008871.2	Mm00435860_m1	heart, kidney	[28]
**31**	CYP7A1	cytochrome P450, family 7a1	NM_007824.2	Mm00484152_m1	heart, liver	[29]
**32**	Akr1b8	aldo-keto reductase family 1B8	NM_008012.1	Mm00484314_m1	spleen	[12]
**33**	FABP4	fatty acid binding protein 4	NM_024406.2	Mm00445878_m1	spleen	[12]
**34**	Ptpmt1	protein tyrosine phosphatase 1	NM_025576.2	Mm00458631_m1	spleen	[30]
**35**	HINT1	histidine triad nucl. binding prot.	NM_008248.2	Mm00801722_m1	spleen	[30]
**36**	PSMB8	proteasome subunit, beta 8	NM_010724.2	Mm00440207_m1	spleen	[30]
**37**	Hoxa2	homeobox A2	NM_010451.1	Mm00439361_m1	brain	[22]
**38**	DNAJA2	DnaJ (Hsp40) homolog,A2	NM_019794.4	Mm00444898_m1	lung, liver	[7,13]
**39**	OAZI	antizyme inhibitor 1	NM_001102458	Mm00497630_m1	lung	[13]
**40**	SLC25A6	solute carrier family 25A6	NM_026255.5	Mm00470958_m1	lung	[13]
**41**	SERPINCI	serpin peptidase inhibitor, C1	NM_000488.3	Mm00446573_m1	lung	[13]
**42**	HSPCB	heat shock protein 90 alpha B1	NM_008302.3	Mm00833431_g1	lung	[13]
**43**	UBC	ubiquitin C	NM_019639.4	Mm01201237_m1	lung	[13]
**44**	TIMP2	tissue inhib. metalloprot. 2	NM_011594.3	Mm00441825_m1	lung	[13]
**45**	FAS	Fas (TNF receptor superfamily 6)	NM_001146708	Mm01204974_m1	liver	[31,32]
**46**	PCNA	proliferating cell nuclear antigen	NM_011045.2	Mm00448100_g1	liver	[7,11]
**47**	PRDX1	peroxiredoxin 1	NM_011034.4	Mm01621996_s1	liver, lung	[7]
**48**	Ephx1	epoxide hydrolase 1	NM_010145.2	Mm00468752_m1	spleen	[7]
**49**	Hspa1a	heat shock protein 1A	NM_010479.2	Mm01159846_s1	liver	[7]
**50**	SOD1	superoxide dismutase 1	NM_011434.1	Mm01344233_g1	liver, heart	[7]
**51**	Ftl1	ferritin light chain 1	NM_010240.2	Mm03030144_g1	liver, spleen	[12]
**52**	Nqo1	NAD(P)H dehydrogenase 1	NM_008706.5	Mm00500821_m1	liver	[7]
**53**	c-Fos	FBJ osteosarcoma oncogene	NM_010234.2	Mm00487425_m1	liver	[7,21]
**54**	PPIA	peptidylprolyl isomerase A	NM_008907.1	Mm02342430_g1	control	-
**55**	PGK1	phosphoglycerate kinase 1	NM_000291.3	Mm00435617_m1	control	-
**56**	RPLP0	ribosomal protein, large, P0	NM_007475.5	Mm00725448_s1	control	-

## Abbreviations

QRT-PCR: quantitative real-time PCR
ADME: absorption, distribution, metabolism and excretion
