# Supplementary material for: High-Density Real-Time PCR-Based in Vivo Toxicogenomic Screen to Predict Organ-Specific Toxicity
Source: Int J Mol Sci. 2011 Sep 19;12(9):6116–34. doi: 10.3390/ijms12096116 (PMC3189772; doi:10.3390/ijms12096116)

Supplementary Information

Gabriella Fabian [1], Nora Farago [2], Liliana Z. Feher [3], Lajos I. Nagy [3], Sandor Kulin [3], Klara Kitajka [2], Tamas Bito [4], Vilmos Tubak [5], Robert L. Katona [5,6], Laszlo Tiszlavicz [7] and Laszlo G. Puskas [2,3,*]

[1] Avicor Ltd., Közép fasor 52, Szeged H-6726, Hungary; E-Mail: gabriella@avicorbiotech.com
[2] Laboratory of Functional Genomics, Institute of Genetics, Biological Research Center, Hungarian Academy of Sciences, Temesvári krt. 62, Szeged H-6726, Hungary;
E-Mails: farago.nora@gmail.com (N.F.); klarakitajka@gmail.com (K.K.)
[3] Avidin Ltd., Közép fasor 52, Szeged H-6726, Hungary;
E-Mails: l.feher@avidinbiotech.com (L.Z.F.); lajos@avidinbiotech.com (L.I.N.);
kulinsandor@gmail.com (S.K.)
[4] Obstetrics and Gynecology Department, Faculty of Medicine, University of Szeged, Semmelweis u. 1., Szeged H-6725, Hungary; E-Mail: bito@obgyn.szote.u-szeged.hu
[5] Curamach Ltd., Temesvári krt. 62, Szeged H-6726, Hungary; E-Mails: vili@brc.hu (V.T.); katona@brc.hu (R.L.K.)
[6] Laboratory of Chromosome Structure and Function, Institute of Genetics, Biological Research Center, Hungarian Academy of Sciences, Temesvári krt. 62, Szeged H-6726, Hungary
[7] Department of Pathology, University of Szeged, Szeged H-6725, Hungary;
E-Mail: tiszlats@patho.szote.u-szeged.hu

* Author to whom correspondence should be addressed; E-Mail: laszlo@avidinbiotech.com;
Tel.: +36-62-546-973; Fax: +36-62-546-972.

Received: 29 April 2011; in revised form: 24 August 2011 / Accepted: 5 September 2011 / Published: 19 September 2011

Abstract: Toxicogenomics, based on the temporal effects of drugs on gene expression, is able to predict toxic effects earlier than traditional technologies by analyzing changes in genomic biomarkers that could precede subsequent protein translation and initiation of histological organ damage. In the present study our objective was to extend *in vivo* toxicogenomic screening from analyzing one or a few tissues to multiple organs, including heart, kidney, brain, liver and spleen. Nanocapillary quantitative real-time PCR (QRT-PCR) was used in the study, due to its higher throughput, sensitivity and reproducibility, and larger dynamic range compared to DNA microarray technologies. Based on previous data, 56 gene markers were selected coding for proteins with different functions, such as proteins for acute phase response, inflammation, oxidative stress, metabolic processes, heat-shock response, cell cycle/apoptosis regulation and enzymes which are involved in detoxification. Some of the marker genes are specific to certain organs, and some of them are general indicators of toxicity in multiple

organs. Utility of the nanocapillary QRT-PCR platform was demonstrated by screening different references, as well as discovery of drug-like compounds for their gene expression profiles in different organs of treated mice in an acute experiment. For each compound, 896 QRT-PCR were done: four organs were used from each of the treated four animals to monitor the relative expression of 56 genes. Based on expression data of the discovery gene set of toxicology biomarkers the cardio- and nephrotoxicity of doxorubicin and sulfasalazin, the hepato- and nephrotoxicity of rotenone, dihydrocoumarin and aniline, and the liver toxicity of 2,4-diaminotoluene could be confirmed. The acute heart and kidney toxicity of the active metabolite SN-38 from its less toxic prodrug, irinotecan could be differentiated, and two novel gene markers for hormone replacement therapy were identified, namely *fabp4* and *pparg*, which were down-regulated by estradiol treatment.

Keywords: toxicogenomics; organ toxicity; real-time PCR; gene expression

---

Supplementary Figure 1

Control liver

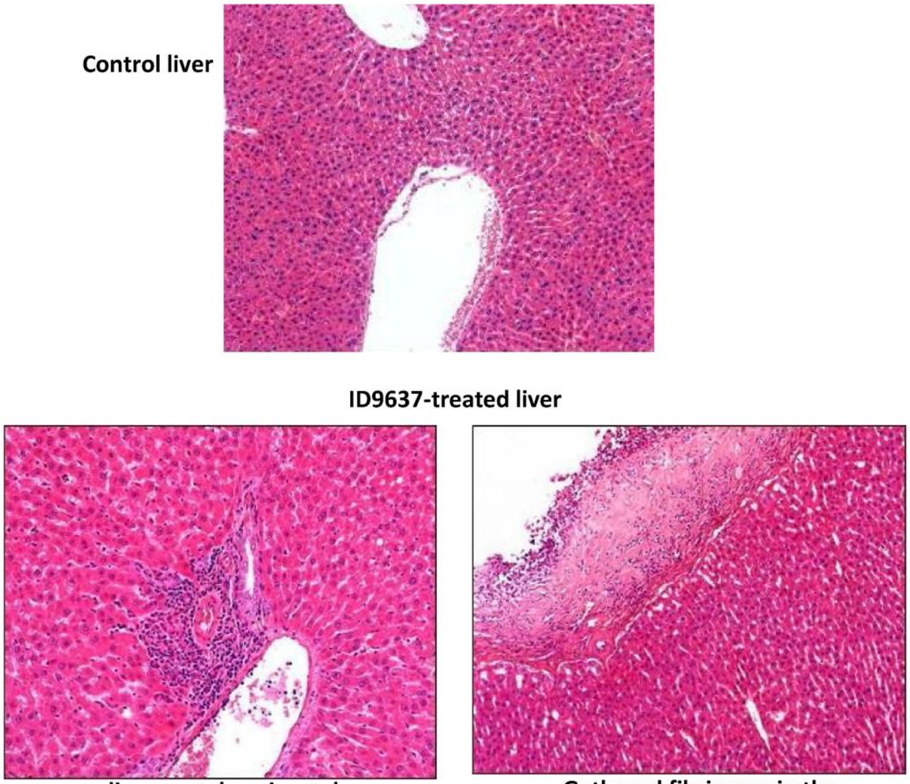

ID9637-treated liver

medium portal-periportal inflammation

Gathered fibrin-pus in the fibrotic liver capsule

Supplementary Figure 2

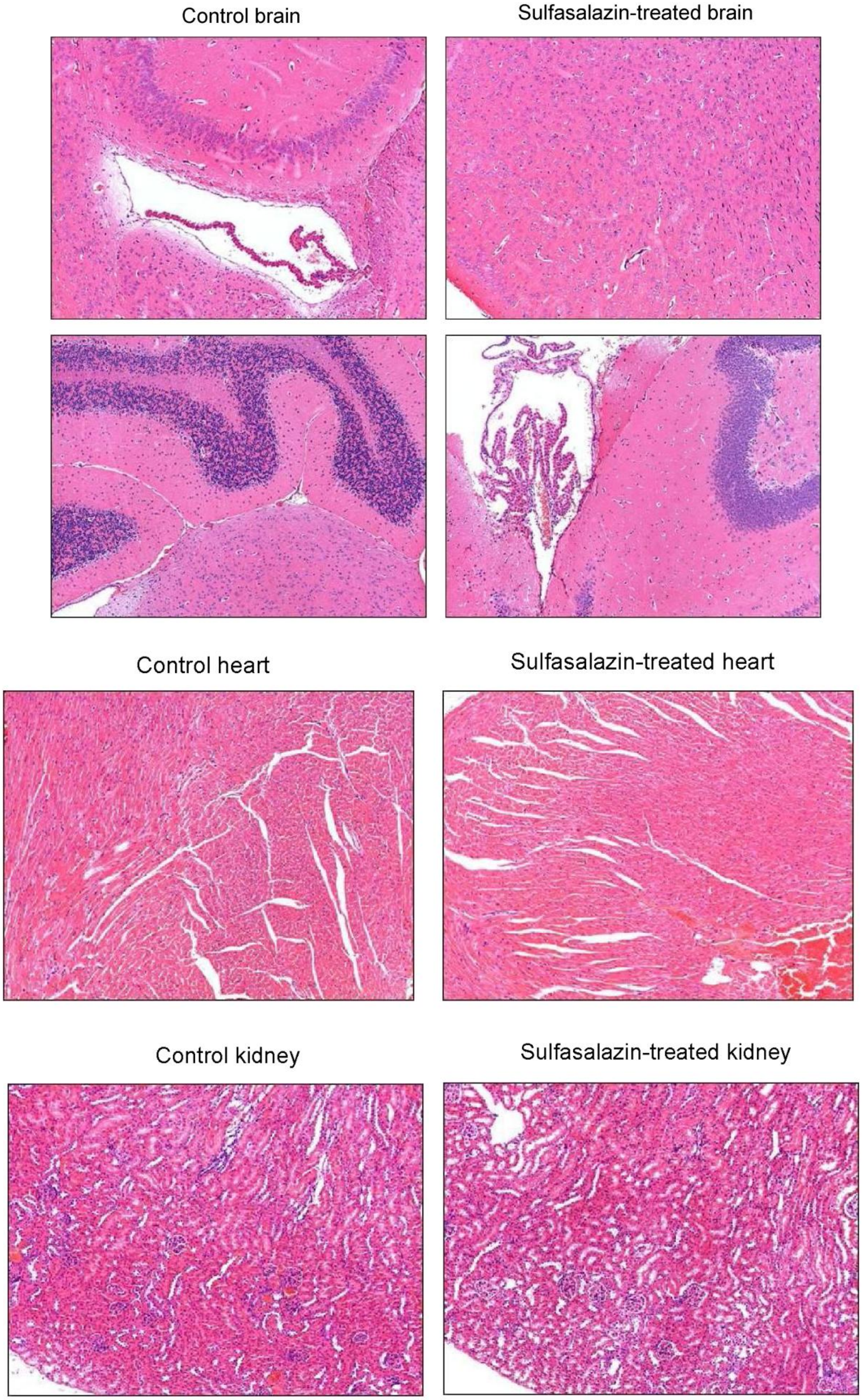

Control liver

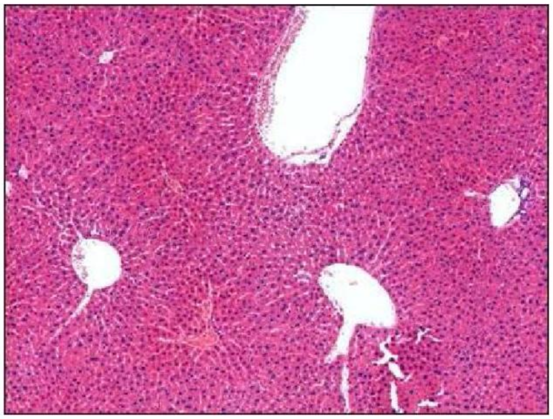

Sulfasalazin-treated liver

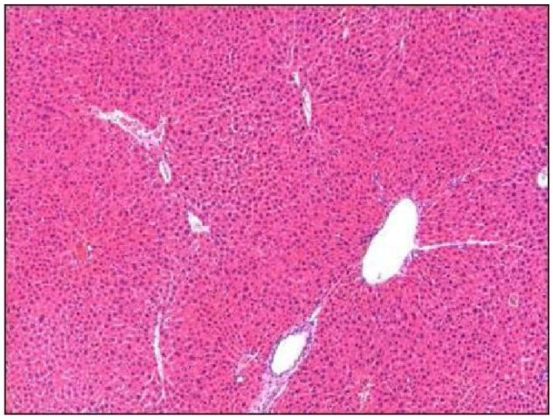

Control lungs

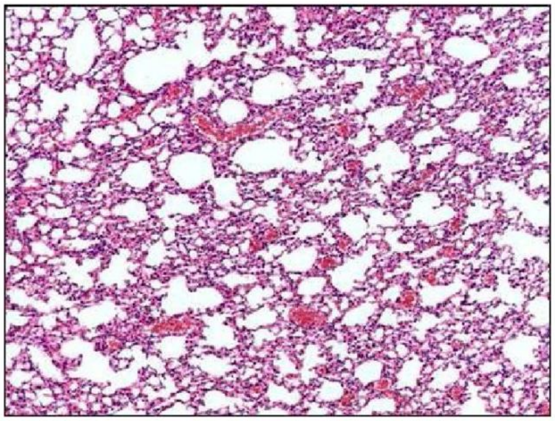

Sulfasalazin-treated lungs

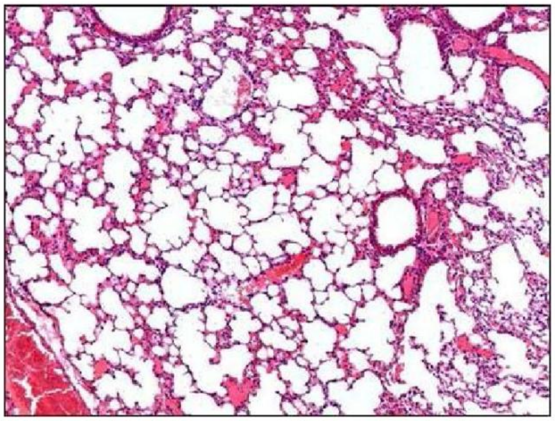

Control spleen

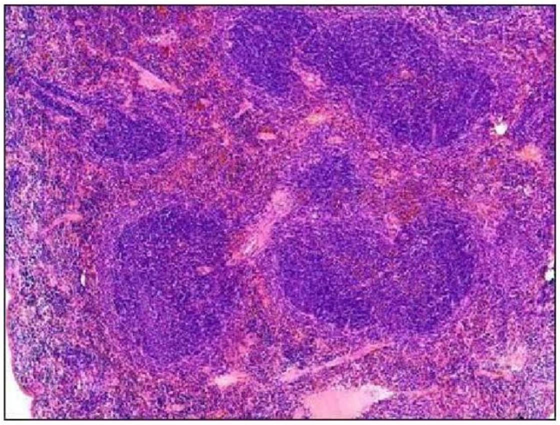

Sulfasalazin-treated spleen

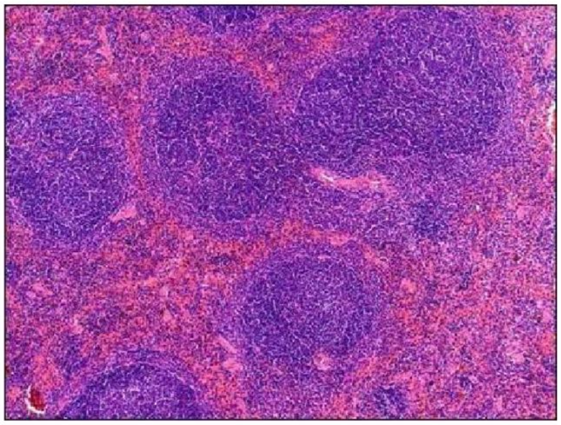

Supplement: Supplementary file 1 [file ijms-12-06116-s001.zip › ijms-12-06116-Supplementary Figures.pdf]
