# Supplementary material for: High-Density Real-Time PCR-Based in Vivo Toxicogenomic Screen to Predict Organ-Specific Toxicity
Source: Int J Mol Sci. 2011 Sep 19;12(9):6116–34. doi: 10.3390/ijms12096116 (PMC3189772; doi:10.3390/ijms12096116)
Supplement: Supplementary file 1 [file ijms-12-06116-s001.zip › ijms-12-06116-Supplementary Table 1.pdf]

NAME	Doxo B	Doxo L	Doxo H	Doxo K	Sulfa B	Sulfa L	Sulfa H	Sulfa K
PSMB8	#DIV/0!	0.394944	0.056412	0.181726	0.297095	0.240653	0.125602	#DIV/0!
Clu	0.793136	0.264324	0.664902	0.212571	0.774687	0.290549	0.46847	0.481647
Reg3a	#DIV/0!	#DIV/0!	#DIV/0!	#DIV/0!	#DIV/0!	#DIV/0!	#DIV/0!	#DIV/0!
PEPCK	0.623068	0.324206	0.165463	0.232414	#DIV/0!	0.391947	#DIV/0!	0.350465
PPIA, pep	0.613314	0.469628	0.614187	0.357245	0.601617	0.169499	0.315283	0.702852
PCNA	0.583648	0.499722	0.667859	0.206852	0.544555	0.469976	0.25195	0.352062
PRDX1	0.682315	0.275811	0.429499	0.327521	0.681865	0.177189	0.327185	0.209563
NOX3	#DIV/0!	#DIV/0!	#DIV/0!	#DIV/0!	#DIV/0!	#DIV/0!	#DIV/0!	#DIV/0!
Ptpmt1	0.645924	0.705713	0.587348	0.476082	0.660503	0.225492	0.193019	0.559362
Klk1b3	#DIV/0!	#DIV/0!	#DIV/0!	1.6333	#DIV/0!	#DIV/0!	#DIV/0!	0.175362
Gstp2	0.684582	0.715137	1.131124	0.524766	0.460327	0.682208	0.42998	0.052326
Odc1	0.761913	0.197999	0.661105	0.395603	0.764663	0.210833	0.270878	0.264321
vim	0.526589	0.546008	1.029442	0.813654	0.301253	0.69248	0.202045	0.390027
Pgam2	0.745157	#DIV/0!	0.52542	0.571972	0.724731	#DIV/0!	0.199727	0.450376
Prdx3	0.707955	0.510262	0.570867	0.631552	0.711886	0.340529	0.209557	0.410073
SLC25A6	0.849638	0.33886	0.569395	0.127441	0.756168	0.471736	0.506848	0.142149
PGK1, pho	0.315819	0.348646	0.519183	0.459047	0.305738	0.214597	0.132116	0.490399
SERPINE1	#DIV/0!	0.736741	0.875154	0.467543	0.32356	0.717148	0.351304	0.436992
DNAJA2	0.785478	0.459606	0.585967	0.12774	0.769329	0.309088	0.093677	0.23062
Egf	0.277847	#DIV/0!	0.647063	0.207735	0.250868	0.112527	0.653399	0.4454
Tubb5	0.893185	0.643913	0.812062	0.378862	0.185564	0.527179	0.29786	0.982968
Idh3B	0.720769	0.280871	0.726824	0.438429	0.662956	0.294491	0.173518	0.502717
Hspa1a	0.723419	1.024804	0.336996	0.830053	0.751115	0.301904	0.610576	0.803824
GADD153	0.640223	0.731784	0.695501	0.585305	0.605573	0.719097	0.566811	0.452504
Hsd3b4	0.221324	#DIV/0!	#DIV/0!	0.247402	0.257216	0.120208	#DIV/0!	0.398317
HSPCB	0.887158	0.71444	0.34261	0.617396	0.781967	0.367494	0.272342	0.744612
TIMP3	0.668982	0.740342	0.556382	0.221612	0.656955	0.698629	0.252931	0.321676
Cyp1a1	#DIV/0!	#DIV/0!	#DIV/0!	0.387945	#DIV/0!	#DIV/0!	#DIV/0!	#DIV/0!
SAA3	0.101823	0.87548	0.758595	0.221324	0.415093	0.856595	#DIV/0!	#DIV/0!
CYP7A1	#DIV/0!	0.764063	#DIV/0!	#DIV/0!	#DIV/0!	0.835371	#DIV/0!	#DIV/0!
Ccng1	0.40499	0.488596	0.211711	0.560409	0.413122	0.391033	0.173727	0.61854
Alox12b	0.43768	#DIV/0!	#DIV/0!	#DIV/0!	0.407765	#DIV/0!	#DIV/0!	#DIV/0!
Akr1b8	#DIV/0!	0.388174	0.2725	#DIV/0!	#DIV/0!	0.780453	0.167106	0.720541
c-Fos	0.816205	0.910215	0.745112	#DIV/0!	0.702994	0.492748	0.679759	0.438348
Hoxa2	#DIV/0!	0.282136	0.853478	0.605619	#DIV/0!	#DIV/0!	0.91555	0.610039
Spp1	0.475888	0.728489	0.420945	0.534024	0.689459	0.503023	0.028284	0.443471
Fga	0.725492	0.665095	0.643467	0.454047	0.343654	0.616138	#DIV/0!	0.713095
UBC	0.360868	0.619343	0.322075	0.416279	0.463395	0.507502	0.295371	0.583194
Nqo1	0.636116	0.820588	0.45071	0.168335	0.646778	0.719253	0.2829	0.324996
Aadat	0.423557	0.617515	#DIV/0!	0.268609	0.551543	0.385698	#DIV/0!	0.26587
Kap	#DIV/0!	0.307591	#DIV/0!	0.140113	0.364737	0.307591	#DIV/0!	0.612518
Oat	0.372179	0.303501	0.822107	0.395811	0.747171	0.391768	0.281895	0.362579
Hsd17b4	0.646387	0.406652	0.312258	0.831488	0.646888	0.354818	0.299552	0.430771
RPLP0, rib	0.568126	0.745998	0.395426	0.065054	0.705855	0.887893	0.391202	0.402128
FAS	0.483106	0.349841	0.522855	0.489908	0.326335	0.628272	0.353915	0.611033
Ndufa5	0.485859	0.281765	0.131549	0.246182	0.366219	0.174408	0.137245	0.181019
SOD1	0.367166	0.442872	0.348219	0.356354	0.112001	0.18155	0.353289	0.295946
SERPINCI	0.523259	0.243552	#DIV/0!	0.393418	0.730122	0.29486	#DIV/0!	0.847814
	#DIV/0!	#DIV/0!	#DIV/0!	#DIV/0!	#DIV/0!	#DIV/0!	#DIV/0!	#DIV/0!
	#DIV/0!	#DIV/0!	#DIV/0!	#DIV/0!	#DIV/0!	#DIV/0!	#DIV/0!	#DIV/0!
Ephx1	0.766063	0.551667	0.791697	0.163645	0.599449	0.43452	0.245233	0.343052
Ftl1	0.571997	0.458211	0.860842	0.230071	0.573866	0.199029	0.319872	0.116543
Anxa2	0.551223	0.555888	0.196297	0.34174	0.514283	0.637788	0.217417	0.394091
Rbp4	0.781812	0.289386	0.763276	0.868691	0.584943	0.358568	0.872517	0.728412

	#DIV/0!	#DIV/0!	#DIV/0!	#DIV/0!	#DIV/0!	#DIV/0!	#DIV/0!	#DIV/0!
	#DIV/0!	#DIV/0!	#DIV/0!	#DIV/0!	#DIV/0!	#DIV/0!	#DIV/0!	#DIV/0!
	#DIV/0!	#DIV/0!	#DIV/0!	#DIV/0!	#DIV/0!	#DIV/0!	#DIV/0!	#DIV/0!
	#DIV/0!	#DIV/0!	#DIV/0!	#DIV/0!	#DIV/0!	#DIV/0!	#DIV/0!	#DIV/0!
HINT1	0.753955	0.550828	0.38126	0.370582	0.121499	0.309288	0.434196	0.420049
OAZI	0.739689	0.693621	0.684323	0.61673	0.189466	0.418376	0.503062	0.604555
FABP4	0.492419	0.394226	0.935961	0.237862	0.06481	0.673201	0.133809	0.610682
TIMP2	0.263606	0.866044	0.724242	0.282652	0.614425	0.729902	0.371877	0.241274
	#DIV/0!							
	#DIV/0!							

Rot B	Rot L	Rot H	Rot K	Anilin B	Anilin L	Anilin H	Anilin K	ID9637 B
0.497712	0.148773	#DIV/0!	#DIV/0!	0.794416	0.214746	0.860434	#DIV/0!	0.674275
0.350018	0.302219	0.191906	0.284592	0.710847	0.51502	0.521163	0.536343	0.781308
#DIV/0!	#DIV/0!	#DIV/0!	#DIV/0!	#DIV/0!	#DIV/0!	#DIV/0!	#DIV/0!	#DIV/0!
0.826204	0.371499	#DIV/0!	0.35499	0.836527	0.291539	0.85616	0.654144	0.827343
0.287736	0.275359	0.183443	0.437961	0.654582	0.327168	0.422986	2.170664	0.730054
0.103575	0.518805	0.18404	0.303329	0.888744	0.644249	0.352797	0.228229	0.658117
0.354508	0.176521	0.157584	0.503001	0.639613	0.299167	0.510279	0.307129	0.677114
#DIV/0!	#DIV/0!	#DIV/0!	#DIV/0!	#DIV/0!	#DIV/0!	#DIV/0!	#DIV/0!	#DIV/0!
0.334175	0.135193	0.214137	0.541727	0.604539	0.151361	0.330767	0.486552	0.783094
#DIV/0!	#DIV/0!	#DIV/0!	0.285089	#DIV/0!	#DIV/0!	#DIV/0!	0.92631	#DIV/0!
#DIV/0!	0.47058	#DIV/0!	0.405172	1.6555	0.897791	0.421453	#DIV/0!	0.258094
0.480581	0.210271	0.210071	0.476896	0.677349	0.160106	0.36342	0.240901	0.772991
0.733069	0.565399	0.262289	0.409558	0.448994	0.59501	0.316278	0.874712	0.3703
0.652736	#DIV/0!	#DIV/0!	0.475538	0.93921	#DIV/0!	0.237868	0.877894	0.815509
0.628666	0.332992	0.168459	0.57721	0.748948	0.40531	0.237875	0.309163	0.729444
0.713261	0.308696	0.226566	0.699059	0.887419	0.612994	0.425901	0.380913	#DIV/0!
0.304038	0.255343	0.131074	0.429571	0.609047	0.270121	0.259572	0.452965	0.318535
0.956008	0.762904	0.234001	0.381474	0.535538	0.926435	0.408107	0.829109	#DIV/0!
0.35535	0.425437	0.216657	0.291531	0.648274	0.417605	0.182823	0.199067	0.814019
0.269408	#DIV/0!	#DIV/0!	0.200696	#DIV/0!	0.006364	0.693713	0.467088	0.269408
0.270062	0.512334	0.264139	0.379481	0.153185	0.734279	0.690317	0.391415	0.60243
0.299355	0.23392	0.194389	0.42905	0.740997	0.218915	0.442493	0.47046	0.660764
0.416992	0.967731	0.26892	0.827924	0.715531	0.795726	0.357956	1.168002	0.774988
0.338599	0.771602	0.141888	0.661101	0.803807	0.794322	0.464897	0.719998	0.673989
0.893428	0.924423	#DIV/0!	0.295236	0.352908	0.398101	0.445501	0.563946	0.206475
0.526489	0.462604	0.188172	0.663165	0.936511	0.581739	0.300836	0.641207	0.793615
0.605343	0.643607	0.200861	0.313095	0.742757	0.704635	0.110681	0.367859	0.692388
#DIV/0!	#DIV/0!	#DIV/0!	#DIV/0!	#DIV/0!	#DIV/0!	#DIV/0!	#DIV/0!	#DIV/0!
#DIV/0!	0.999109	#DIV/0!	#DIV/0!	0.408096	0.86578	0.135057	0.937624	0.881645
#DIV/0!	0.881916	#DIV/0!	#DIV/0!	#DIV/0!	0.831903	#DIV/0!	#DIV/0!	#DIV/0!
0.384414	0.470951	0.146678	0.539509	0.391346	0.453382	0.323836	0.563126	0.476678
0.837279	#DIV/0!	#DIV/0!	#DIV/0!	0.754207	#DIV/0!	#DIV/0!	#DIV/0!	0.91609
#DIV/0!	0.364311	#DIV/0!	#DIV/0!	0.729941	0.348239	0.566596	0.730198	#DIV/0!
0.97597	1.06801	#DIV/0!	0.515407	0.805394	0.922665	0.591199	0.041719	0.76146
#DIV/0!	#DIV/0!	#DIV/0!	0.794422	#DIV/0!	#DIV/0!	0.849854	#DIV/0!	#DIV/0!
0.757606	0.727149	0.231005	0.43305	0.724967	0.809177	0.028284	0.586861	0.885992
0.793397	0.42968	#DIV/0!	0.569382	0.792169	0.448153	0.643467	1.245044	0.982196
0.973587	0.312627	0.212864	0.518573	0.504102	0.498838	0.367329	0.403089	0.365159
0.515826	0.717016	0.225707	0.429393	0.93466	0.88873	0.567272	0.427865	0.573338
0.577786	0.219875	#DIV/0!	0.272011	0.989816	0.381144	#DIV/0!	0.772466	0.372645
1.133157	#DIV/0!	#DIV/0!	0.284469	0.019092	0.54256	#DIV/0!	0.257108	#DIV/0!
0.377296	0.458454	0.192	0.27912	0.783564	0.464353	0.259038	0.200183	0.756004
0.586867	0.364908	0.180075	0.498216	0.854171	0.606564	0.322088	0.39822	0.927284
0.712057	0.719356	0.259168	0.277186	1.919826	0.697779	0.512629	0.941809	0.867424
0.705198	0.395396	0.139183	0.278945	0.175948	0.943012	0.705807	0.58132	0.684867
#DIV/0!	0.142619	#DIV/0!	0.618928	0.420021	0.170872	0.863745	0.798986	1.417042
0.407893	0.217382	0.109877	0.471824	0.794977	0.257969	0.648971	0.570143	0.784321
0.202233	0.271564	#DIV/0!	0.74776	0.533995	0.249071	0.435578	0.781193	1.191262
#DIV/0!	#DIV/0!	#DIV/0!	#DIV/0!	#DIV/0!	#DIV/0!	#DIV/0!	#DIV/0!	#DIV/0!
#DIV/0!	#DIV/0!	#DIV/0!	#DIV/0!	#DIV/0!	#DIV/0!	#DIV/0!	#DIV/0!	#DIV/0!
0.617521	0.425661	0.197492	0.230121	0.894251	0.530575	0.237587	0.413808	0.769257
0.331228	0.117704	0.242208	0.369908	0.671863	0.332395	0.075256	0.32743	0.613609
0.630911	0.521226	0.166409	0.415343	0.832758	0.534907	0.598392	0.612159	0.514847
0.713346	0.327889	0.231901	0.725263	0.665826	0.396497	0.756703	1.219122	0.877931

#DIV/0!	#DIV/0!	#DIV/0!	#DIV/0!	#DIV/0!	#DIV/0!	#DIV/0!	#DIV/0!	#DIV/0!
#DIV/0!	#DIV/0!	#DIV/0!	#DIV/0!	#DIV/0!	#DIV/0!	#DIV/0!	#DIV/0!	#DIV/0!
#DIV/0!	#DIV/0!	#DIV/0!	#DIV/0!	#DIV/0!	#DIV/0!	#DIV/0!	#DIV/0!	#DIV/0!
#DIV/0!	#DIV/0!	#DIV/0!	#DIV/0!	#DIV/0!	#DIV/0!	#DIV/0!	#DIV/0!	#DIV/0!
0.300859	0.166946	0.133478	0.542068	1.071509	0.107273	0.478647	0.541757	0.813231
0.150443	0.357094	0.203986	0.666126	0.819422	0.207101	0.242469	0.600374	0.67764
0.462448	0.432216	0.27557	0.367867	#DIV/0!	0.726728	0.332408	0.528629	0.707814
0.664616	0.65846	0.219537	0.331883	0.895163	0.800173	0.248446	0.285264	0.694855

ID9637 L	ID9637 H	ID9637 K	DHC B	DHC L	DHC H	DHC K	DATol B	DATol L
0.263089	0.066114	0.519878	0.80609	0.368415	0.06082	0.507165	0.810344	0.282118
0.339811	0.504908	0.184717	0.43588	0.26361	0.50496	0.519981	0.393996	0.399951
#DIV/0!	#DIV/0!	#DIV/0!	#DIV/0!	#DIV/0!	#DIV/0!	#DIV/0!	#DIV/0!	#DIV/0!
0.455224	0.165463	0.481238	0.83807	0.342688	0.935872	0.868699	0.738514	0.48832
0.257276	0.316404	0.341781	0.648284	0.220357	0.596421	0.581902	0.297979	0.709827
0.458062	0.386689	0.231512	0.695379	0.463851	0.652845	0.46229	0.225482	0.690513
0.219715	0.451394	0.202189	0.799853	0.145335	0.638843	0.427329	0.273546	0.143133
#DIV/0!	#DIV/0!	#DIV/0!	#DIV/0!	#DIV/0!	#DIV/0!	#DIV/0!	#DIV/0!	#DIV/0!
0.495549	0.590224	0.717393	0.733679	0.382102	0.474174	0.742576	0.347386	0.726604
#DIV/0!	#DIV/0!	#DIV/0!	#DIV/0!	#DIV/0!	#DIV/0!	0.715134	#DIV/0!	#DIV/0!
0.506757	0.516011	#DIV/0!	#DIV/0!	0.493213	0.392535	0.481099	#DIV/0!	0.469579
0.361925	0.318413	0.393983	0.781662	0.207808	0.723729	0.467153	0.56762	0.193384
0.635314	0.42428	0.778229	0.326309	0.551218	0.662606	1.116953	0.363035	0.793757
#DIV/0!	0.369483	0.555013	0.716201	#DIV/0!	0.635986	0.615792	0.893698	#DIV/0!
0.353413	0.244839	0.30036	0.767489	0.358289	0.640905	0.643569	0.779916	0.453246
0.263546	0.414386	0.39503	0.845376	0.489125	0.976617	0.763777	0.695288	0.840212
0.203351	0.540959	0.479885	0.377283	0.281423	0.791761	0.733633	0.398515	0.654851
0.81867	0.783937	0.940243	0.836507	0.881601	0.544177	1.174258	0.363016	0.731687
0.31033	0.257167	0.252184	0.467177	0.328933	0.55416	0.606737	0.369761	0.869308
0.458205	0.738996	0.234646	0.872906	0.631975	0.72289	0.838806	#DIV/0!	0.159806
0.531777	0.345746	0.590351	0.21146	0.450977	0.749014	0.961323	0.369116	0.626355
0.216557	0.16742	0.416729	0.424285	0.188717	0.51492	0.62523	0.388977	0.526912
0.432472	0.148492	0.910423	0.78349	0.769596	0.359917	0.893368	0.598125	0.562645
0.757641	0.54174	0.482175	0.745494	0.783468	0.680788	0.479588	0.733399	0.778521
0.070004	#DIV/0!	0.452298	0.233345	0.884096	#DIV/0!	0.41606	0.509632	0.901584
0.385684	0.398446	0.619613	0.811053	0.402329	0.524102	0.970602	0.587816	0.43689
0.677025	0.244839	0.266249	0.701362	0.836538	0.823384	0.711303	0.311415	0.791286
#DIV/0!	#DIV/0!	#DIV/0!	#DIV/0!	#DIV/0!	#DIV/0!	0.115258	#DIV/0!	#DIV/0!
0.757232	0.469248	0.780646	#DIV/0!	0.645747	0.295571	#DIV/0!	0.732563	0.440986
0.608852	#DIV/0!	#DIV/0!	#DIV/0!	0.779582	#DIV/0!	#DIV/0!	#DIV/0!	1.15832
0.565893	0.132997	0.599148	0.399082	0.424733	0.370159	0.726496	0.314432	0.582066
#DIV/0!	#DIV/0!	#DIV/0!	0.562764	#DIV/0!	#DIV/0!	#DIV/0!	0.666076	#DIV/0!
0.573309	0.167106	0.696351	#DIV/0!	0.226981	0.523504	0.707814	#DIV/0!	0.667408
0.986649	0.622586	0.390743	0.858063	0.812141	0.672097	0.64506	0.755779	0.915968
#DIV/0!	0.891198	0.663783	#DIV/0!	#DIV/0!	0.76604	0.633899	#DIV/0!	#DIV/0!
0.917971	0.028284	0.394393	0.81416	0.746364	1.91659	0.412408	0.691823	0.784221
0.383107	0.785328	0.790268	2.673469	0.433545	0.643467	0.675841	0.725492	0.452023
0.405655	0.546155	0.474388	1.085207	0.391012	0.443937	0.436686	0.054556	0.657568
0.711559	0.498442	0.330037	0.805968	0.79058	0.504105	0.694669	0.60362	0.73434
0.250906	#DIV/0!	0.224336	0.258801	0.286225	#DIV/0!	0.497484	0.483615	0.371044
0.547301	#DIV/0!	0.223261	0.269408	#DIV/0!	#DIV/0!	0.482062	#DIV/0!	0.307591
0.840722	0.295647	0.114199	0.563166	0.400939	0.646335	0.444328	0.360109	0.28861
0.475359	0.408853	0.387742	0.956721	0.400841	0.825353	0.666288	0.731175	0.718669
0.662559	0.81469	0.940363	0.707073	0.915855	0.479658	#DIV/0!	0.621405	0.548423
0.5603	0.422406	0.48164	0.839128	0.462801	0.499145	0.417116	0.144931	0.558643
0.209604	0.165505	0.781963	0.482818	0.140209	0.562657	0.159751	0.676892	0.199292
0.199719	0.365191	0.269311	0.496799	0.240047	0.400809	0.575036	0.385034	0.226354
0.311978	#DIV/0!	0.025658	0.419935	0.247886	#DIV/0!	0.479418	0.523259	0.345937
#DIV/0!	#DIV/0!	#DIV/0!	#DIV/0!	#DIV/0!	#DIV/0!	#DIV/0!	#DIV/0!	#DIV/0!
#DIV/0!	#DIV/0!	#DIV/0!	#DIV/0!	#DIV/0!	#DIV/0!	#DIV/0!	#DIV/0!	#DIV/0!
0.824188	0.22555	0.28452	0.604571	0.417549	0.578781	0.593012	0.791337	0.562934
0.215243	0.419463	0.219458	0.635119	0.305878	0.598997	0.520612	0.451609	0.397526
0.567546	0.519448	0.561247	0.485898	0.508231	0.643883	0.614855	0.511603	0.838526
0.597111	0.875926	0.294968	0.510519	0.277577	0.88008	0.590636	0.443683	0.351284

#DIV/0!	#DIV/0!	#DIV/0!	#DIV/0!	#DIV/0!	#DIV/0!	#DIV/0!	#DIV/0!	#DIV/0!
#DIV/0!	#DIV/0!	#DIV/0!	#DIV/0!	#DIV/0!	#DIV/0!	#DIV/0!	#DIV/0!	#DIV/0!
#DIV/0!	#DIV/0!	#DIV/0!	#DIV/0!	#DIV/0!	#DIV/0!	#DIV/0!	#DIV/0!	#DIV/0!
#DIV/0!	#DIV/0!	#DIV/0!	#DIV/0!	#DIV/0!	#DIV/0!	#DIV/0!	#DIV/0!	#DIV/0!
0.154046	0.238158	0.432825	0.878762	0.257999	0.491801	0.653679	0.264996	0.504062
0.195337	0.445665	0.60732	0.743223	0.371148	0.791654	0.898235	0.110856	0.326984
0.390824	0.143414	0.433293	#DIV/0!	0.507185	0.422327	0.314249	0.777091	0.439265
0.683833	0.367542	0.22246	0.629732	0.781391	0.921097	0.720821	0.767177	0.671562

DATol H	DATol K
0.317792	0.754997
0.409556	0.315562
#DIV/0!	#DIV/0!
0.764164	0.482719
0.356319	0.346775
0.292311	0.317173
0.318934	0.254866
#DIV/0!	#DIV/0!
0.165266	0.493308
#DIV/0!	0.310696
0.511011	0.91444
0.268054	0.5432
0.186209	0.838174
0.273246	0.488142
0.195396	0.366196
0.519845	0.397262
0.218136	0.44232
0.221923	0.911943
0.141159	0.372599
0.667558	0.36976
0.32073	0.950946
0.202388	0.477402
#DIV/0!	0.920637
0.54236	0.252416
0.763366	0.315568
0.162211	0.694321
0.069586	0.405738
#DIV/0!	#DIV/0!
0.706379	0.583988
#DIV/0!	#DIV/0!
0.16619	0.611854
#DIV/0!	#DIV/0!
0.657539	0.95813
0.812216	0.364338
0.990082	0.736098
0.253932	0.479551
0.643467	0.713667
0.224069	0.444279
0.27716	0.401568
#DIV/0!	0.296867
#DIV/0!	0.477447
0.218902	0.385793
0.354275	0.380756
0.391202	0.591141
0.238638	0.407038
0.156417	0.894487
0.388578	0.258801
#DIV/0!	0.709935
#DIV/0!	#DIV/0!
#DIV/0!	#DIV/0!
0.142146	0.398792
0.32031	0.249523
0.237742	0.357395
0.894827	0.415552

#DIV/0!	#DIV/0!
#DIV/0!	#DIV/0!
#DIV/0!	#DIV/0!
#DIV/0!	#DIV/0!
0.268087	0.432986
0.445738	0.633423
0.319288	0.371234
0.323355	0.638816
